# Ultrastructure and Viral Metagenome of Bacteriophages from an Anaerobic Methane Oxidizing *Methylomirabilis* Bioreactor Enrichment Culture

**DOI:** 10.3389/fmicb.2016.01740

**Published:** 2016-11-08

**Authors:** Lavinia Gambelli, Geert Cremers, Rob Mesman, Simon Guerrero, Bas E. Dutilh, Mike S. M. Jetten, Huub J. M. Op den Camp, Laura van Niftrik

**Affiliations:** ^1^Department of Microbiology, Faculty of Science, Institute for Water and Wetland Research, Radboud UniversityNijmegen, Netherlands; ^2^Theoretical Biology and Bioinformatics, Utrecht UniversityUtrecht, Netherlands; ^3^Centre for Molecular and Biomolecular Informatics, Radboud University Medical CentreNijmegen, Netherlands

**Keywords:** *Methylomirabilis*, bacteriophage, viral metagenome, ultrastructure, bioreactor

## Abstract

With its capacity for anaerobic methane oxidation and denitrification, the bacterium *Methylomirabilis oxyfera* plays an important role in natural ecosystems. Its unique physiology can be exploited for more sustainable wastewater treatment technologies. However, operational stability of full-scale bioreactors can experience setbacks due to, for example, bacteriophage blooms. By shaping microbial communities through mortality, horizontal gene transfer, and metabolic reprogramming, bacteriophages are important players in most ecosystems. Here, we analyzed an infected *Methylomirabilis* sp. bioreactor enrichment culture using (advanced) electron microscopy, viral metagenomics and bioinformatics. Electron micrographs revealed four different viral morphotypes, one of which was observed to infect *Methylomirabilis* cells. The infected cells contained densely packed ~55 nm icosahedral bacteriophage particles with a putative internal membrane. Various stages of virion assembly were observed. Moreover, during the bacteriophage replication, the host cytoplasmic membrane appeared extremely patchy, which suggests that the bacteriophages may use host bacterial lipids to build their own putative internal membrane. The viral metagenome contained 1.87 million base pairs of assembled viral sequences, from which five putative complete viral genomes were assembled and manually annotated. Using bioinformatics analyses, we could not identify which viral genome belonged to the *Methylomirabilis*- infecting bacteriophage, in part because the obtained viral genome sequences were novel and unique to this reactor system. Taken together these results show that new bacteriophages can be detected in anaerobic cultivation systems and that the effect of bacteriophages on the microbial community in these systems is a topic for further study.

## Introduction

The importance of microorganisms in biogeochemical cycles and global warming is well-known (Falkowski et al., 2008). Particularly, microorganisms that take part in the carbon and nitrogen cycles are of great interest for both the scientific community and the public at large due to the growing awareness of climate change. In fact, among greenhouse gasses that affect the climate, carbon dioxide (CO_2_), methane (CH_4_), and nitrous oxide (N_2_O) are the most relevant (Pachauri et al., 2014).

Aerobic and anaerobic methanotrophic microorganisms oxidize CH_4_ to CO_2_ mitigating the release of CH_4_ to the atmosphere. The newly discovered anaerobic denitrifying methanotroph “*Candidatus Methylomirabilis oxyfera*” was enriched from freshwater sediments and couples methane oxidation to nitrite reduction, thereby linking carbon and nitrogen cycles via a postulated unique intra-aerobic pathway (Ettwig et al., 2009, 2010). Next to its remarkable physiology, *M. oxyfera* stands out from other bacteria also with respect to its cell shape and structure. These Gram-negative, rod-shaped bacteria have a polygonal cell shape constituted by several longitudinal ridges running along the cell length and converging in a cap-like structure at the cell poles (Wu et al., 2012). Furthermore, these microorganisms are not only interesting from a fundamental scientific point of view, but could also be important players in natural ecosystems (Deutzmann et al., 2014; Hu et al., 2014) and be implemented in the removal of dissolved methane and ammonium from digester effluents in combination with anaerobic ammonium oxidizing bacteria (Luesken et al., 2011; Shi et al., 2013).

Bacteria constitute the vast majority of the biomass on Earth (about 90%), yet viruses are the most abundant “biological entities,” comprising ~94% of the nucleic-acid containing-particles (Suttle, 2007). The discovery that viruses indiscriminately occupy marine environments (Suttle, 2005) and freshwater ecosystems (Middelboe et al., 2008) opened a new research area concerning the effect of bacteriophages on microbial populations and the role of bacteriophages in elemental and nutrient cycles. By inducing bacterial lysis, bacteriophages affect abundance, diversity and functioning of microbial populations (Weinbauer and Rassoulzadegan, 2004).

Marine and freshwater viruses show a great variety of different morphologies (Wommack and Colwell, 2000; Sulcius et al., 2011; Borrel et al., 2012). Viruses have a huge heterogeneity in size and genetic structure. The size ranges from 20 to 200 nm in diameter (Brum et al., 2013), and the genetic material can be either dsDNA, ssDNA (+), dsRNA, or ssRNA (±) (Dimmock et al., 2007). Viruses infecting Bacteria and Archaea generally consist of a proteinaceous capsid that contains the genetic material. They can also have a tail, which is used to inject the genetic material into the host. Tailed viruses belong to three families: the *Siphoviridae* (non-contractile, long tail), the *Myoviridae* (contractile, medium-length tail), and the *Podoviridae* (non-contractile, short tail). Tailless viruses have been described with very diverse morphologies, such as polyhedral, filamentous, spindle-like, cubic-like, star-like, or pleomorphic. Among tailless viruses, bacteriophages with an internal lipid bilayer enclosing the genome have been described and assigned to the *Tectiviridae* and *Corticoviridae* families (King et al., 2012).

The striking morphological diversity of viruses is also reflected in their great genetic heterogeneity (Breitbart et al., 2002). Bacteriophages assigned to a certain family often share very little or no sequence similarity. Comparative analysis of bacteriophage genomes often reveals that genes are organized in modules characterized by a different evolutionary history. This phenomenon is known as genome mosaicism, and it is the result of a high degree of horizontal gene exchange (Hatfull, 2008).

The lack of a universal genetic marker and the fact that only a minority of bacterial hosts can be cultivated in laboratories (Edwards and Rohwer, 2005) make the assessment of such heterogeneity very challenging. Although, culture-independent, large-scale metagenomics has paved the way to a more exhaustive understanding of the viral sequence space (Dutilh, 2014), this approach is not flawless. For example, small datasets may be obtained due to the limited yield of viral DNA from environmental samples, biasing downstream analysis. Moreover, viruses show a high spatiotemporal variability (Koskella and Parr, 2015), contributing to a limited overlap between samples. The analysis of viral metagenomes is laborious, partly because of the lack of reference databases and appropriate analytical tools compared to, for example, the ones for microbial metagenomics (Mokili et al., 2012; Hurwitz and Sullivan, 2013). Consequently, a high abundance of unknown viral sequences (65–95%) are reported in new surveys when these metagenomes are mapped against databases of unknown sequences (Breitbart et al., 2007; Mokili et al., 2012). Finally, viral metagenomics studies are frequently still relatively small in scope.

Several studies investigated the complex dynamics of phage-host interactions in lab-scale bioreactor systems (for example Barr et al., 2010; Shapiro et al., 2010). However, there are limited bioreactor studies on virus-host interaction that include viral metagenomics (one example is Kunin et al., 2008). The present paper describes the bacteriophage population in a *Methylomirabilis* bioreactor enrichment culture using (advanced) electron microscopy, viral metagenomics, and bioinformatics analysis. Several viral genomes and morphologies were found and one lytic bacteriophage was observed to infect *Methylomirabilis* cells. Through bioinformatics we attempted to find which of the obtained viral contigs belonged to the bacteriophage that infects *Methylomirabilis* cells. Finally we speculate on the significance of the infection on bacterial growth and population dynamics.

## Materials and methods

### Enrichment conditions

The *Methylomirabilis* enrichment culture (~80% *Methylomirabilis* sp.) was grown in a continuous sequencing batch reactor (Applicon Biotechnology BV, Applisens, Schiedam, the Netherlands) made of stainless steel and glass with a volume of 6 L. The inoculum biomass was derived from a pre-existing enrichment, originally inoculated with sediment samples from a ditch in the Ooijpolder (Ettwig et al., 2009). The culture was kept anoxic by a continuous supply of a gas mixture composed of methane and carbon dioxide (95:5, v/v) and the medium was continuously flushed with a mixture of argon and carbon dioxide (95:5, v/v). The pH was maintained at 7.3 ± 0.1, the temperature was kept stable at 30°C and the system was constantly stirred at 100 RPM. The volume of the enrichment was kept at 4 L by a level sensor controlled pump in sequential cycles of feeding (22.5 h) and rest (30 min to settle, 60 min to pump out excess medium). The HRT (hydraulic retention time) was 4 days and the composition of the medium was (per L) 0.552–2.07 g NaNO_2_ (8–30 mM depending on culture performance), 288 μg MgSO_4_, 192 μg CaCl_2_, 50 μg KH_2_PO_4_, 2.5 mg FeSO_4_*7H_2_O. The trace element solution was adapted from Ettwig et al. (2010) and contained per L: 150 μg ZnSO_4·_7H_2_O, 60 μg CoCl_2_6H_2_O, 400 μg CuSO_4·_, 100μg NiCl_2_6H_2_O, 10 μg H_3_BO_3_, 100 μg MnCl_2_4H_2_O, 10 μg Na_2_WO_4·_2H_2_O, 50 μg Na_2_MoO_4·_2H_2_O, 15 μg SeO_2_, 10 μg CeCl_2_.

### Negative staining of bacteriophages isolated from the *Methylomirabilis* enrichment culture

A 20 ml sample was collected from the bioreactor enrichment culture and filtered with 0.45 and 0.2 μm syringe filters, sequentially (Puradisc Cellulose Acetate, Whatman) to remove microorganisms and keep only the viral fraction. The flow-through was concentrated using spin columns (Vivaspin, GE Healthcare, 10,000 MWCO) at 5000 × g for 5 min at 4°C, (Allegra X-15R Centrifuge, Beckman Coulter) to a final volume of about 60 μl. Approximately 3 μl sample was placed on glow-discharged and carbon-Formvar-coated 100 mesh hexagonal copper grids (Veco) and incubated at room temperature for 15 min. The excess liquid was blotted off using filter paper (Ashless, Grade 589/1 Filtration Paper, Whatman). The specimens were stained with 0.5% uranyl acetate for 1 min, blotted dry, washed in Milli-Q, blotted dry with filter paper and let air dry completely. The virioplankton was observed using a JEOL JEM-2100 transmission electron microscope operated at 200 kV.

### Freeze-etching on the *Methylomirabilis* enrichment culture

A 2 ml sample was collected from the bioreactor enrichment culture and centrifuged at 800 × g for 4 min at 30°C (Microcentrifuge 5417R, Eppendorf, Hamburg, Germany). Supernatant was decanted and the pellet was resuspended in remaining supernatant. For high-pressure freezing, a specimen sandwich was assembled in the specimen holder of a Leica EMHPF. The sandwich consisted of an aluminum spacer ring (0.2 × 3 mm) and a membrane carrier (2.6 × 0.1 mm, no. 16707898) in which 0.2 μl sample was loaded, the sample was sealed off with a gold stub (specimen carrier D3/D2, gold, no. 16770131). After closing the holder, the sample was high-pressure frozen in an EMHPF, operating at 2100 bar. Samples were stored in liquid nitrogen.

To obtain a freeze-fracture replica, the sample was placed in a detachable cold table and loaded onto the stage of a Balzers BAF400 freeze-etch machine, pre-cooled to −170°C. Specimens were fractured at −110°C and subsequently etched for 12 min at the same temperature with a vacuum below 10^−7^ Bar. The specimen was shadowed with 1.3 nm Pt (angle 45°) and 15 nm C (angle 90°) and a replica of the sample was obtained. The biological material underneath the replica was digested in 70% H_2_SO_4_ for 48 h. Replicas were washed twice in MilliQ water and picked up with glow discharged 700 mesh hexagonal copper grids (reference number G276OG, Agar Scientific) and investigated by a JEOL JEM-2100 transmission electron microscope operated at 200 kV.

### Cryofixation, freeze-substitution, epon embedding, thin-sectioning, and post-staining of *Methylomirabilis* enrichment culture

Cells from the bioreactor enrichment culture were harvested and cryofixed by high-pressure freezing (Leica HPM 100; Leica Microsystems, Vienna, Austria). Samples were placed into a 100 μm cavity of a type A platelet (3 mm diameter; 0.1–0.2 mm depth, Leica Microsystems) and closed with the flat side of a lecithin-coated type B platelet (3 mm diameter, 0.3 mm depth). The platelets were stored in liquid nitrogen.

For epon embedding, frozen samples were freeze-substituted in acetone containing 2% OsO_4_, 0.2% uranyl acetate, and 1% H_2_O (Walther and Ziegler, 2002). The substitution followed several intervals: cells were kept at −90°C for 47 h; brought to −60°C at 2°C per hour and kept at −60°C for 8 h; brought to −30°C at 2°C per hour and kept at −30°C for 8 h in a freeze-substitution unit (AFS; Leica Microsystems, Vienna, Austria). To remove uranyl acetate, the samples were washed four times for 30 min in the AFS device at −30°C with acetone containing 2% OsO_4_and 1% H_2_O. Next, fixation was continued on ice for 1 h. OsO_4_ and H_2_O were removed by washing the sample twice in anhydrous acetone for 30 min. Samples were gradually infiltrated with epon resin and polymerized for 72 h at 60°C (Mollenhauer, 1964). Ultrathin sections of 60 nm were cut using a Leica UCT ultramicrotome (Leica Microsystems, Vienna, Austria) and collected on carbon-Formvar-coated 100 mesh hexagonal copper grids (Veco). The sections were then post-stained with 2% uranyl acetate for 20 min and lead citrate for 2 min. After each of the two steps, grids were washed in MilliQ water. Sections were investigated using a JEOL JEM1010 transmission electron microscope operated at 60 kV.

### Electron tomography on infected *Methylomirabilis* cells

Semi-thin sections (200–300 μm) were cut using a diamond knife (Diatome, Biel, Switzerland) and an ultramicrotome (UCT, Leica microsystems) and collected on 50 mesh copper grids with a carbon coated formvar support film. After air drying, grids were post-stained with 4% uranyl acetate in MilliQ for 30 min and Reynolds lead citrate for 2 min. Ten nanometers proteinA gold (CMC, UMC Utrecht, The Netherlands) was applied to the sections to act as fiducial marker during tilt-series acquisition and reconstruction.

Virus infected cells and isolated virus particles were chosen as the region of interest. Dual axis tilt series (−60 to +60) were recorded on the JEOL JEM-2100 microscope operating at 200 kV, using SerialEM for automated image acquisition (Mastronarde, 2005).

Recorded tilt series were reconstructed using the IMOD package (Kremer et al., 1996) and tomograms were generated using both the weighted back-projection and SIRT algorithms. Reconstructed tomograms were segmented by hand using 3DMOD. Iso-surface model was generated from a sub-tomogram employing a circular mask. Summed virtual slices (36 slices) were visualized using the UCSF Chimera package (Pettersen et al., 2004), the histogram was adjusted to fit the density associated with the virus particle.

### Concentration of viral particles by iron chloride and PEG 8000 precipitation

Over a period of ~3 months bioreactor material and effluent (13 L) were collected from the bioreactor enrichment culture and stored at 4°C until further analyses. Since the majority of the microbial population in the bioreactor grows in aggregates, the total sample was split in two: the bioreactor supernatant containing free bacteriophages and the bacterial biomass containing bacteriophages in the aggregates and within the cells. The two samples were obtained by centrifuging the sample at 20,000 × g for 1 h in 350 ml centrifuge tubes (Thermo Scientific, SorvallTM LYNX 4000 centrifuge). The resulting biomass pellets were resuspended in a total volume of 50 mL of the supernatant. Free-floating bacteriophages were precipitated by iron chloride flocculation, whereas bacterial biomass-associated bacteriophages were collected by PEG 8000.

#### Iron chloride flocculation

To isolate free bacteriophages approximately 12.95 L of supernatant was first filtered with 0.45 and 0.2 μm filters sequentially (nuclepore track-etched polycarbonate membrane filters, Whatman) to remove microbial biomass. The sample was subsequently processed by iron chloride flocculation as described previously (John et al., 2011; Cunningham et al., 2015). Since iron is known to be an inhibitor of DNA amplification, it was removed from the sample by extensively washing the sample with MSM buffer (400 mM NaCl, 20 mM MgSO4·Ů7H2O, 50 mM Tris, pH 7.5) in spin columns (Vivaspin, GE Healthcare, 10,000 MWCO) at 2500 × g for 40 min at 4°C, using an Allegra X-15R Centrifuge (Beckman Coulter). The sample was stored at 4°C until further analyses.

#### PEG 8000 precipitation

About 50 ml of microbial biomass was first disrupted by pottering on ice to free the bacteriophages. To digest non-viral DNA and RNA, DNase I (Thermo Scientific, final concentration 1U/ml), and RNase A (Thermo Scientific, final concentration 5U/mL) treatments were simultaneously performed for 1 h at 37°C. The sample volume was divided in 2 ml Eppendorf tubes and PEG 8000 precipitation was performed as described previously (Guo et al., 2012). The sample was centrifuged for 30 min at 2000 × g and the obtained pellet was resuspended in MSM buffer. PEG 8000 was separated from the viral particles as described previously (Colombet and Sime-Ngando, 2012). In addition, to lower the salt concentration, the supernatant containing free phages was extensively washed with MSM buffer in spin columns (Vivaspin, GE Healthcare, 10,000 MWCO) at 2500 × g and 4°C using an Allegra X-15R Centrifuge (Beckman Coulter) to a final concentration of 20 mM KCl. The sample was stored at 4°C until further analyses.

### DNA extraction

The two viral samples obtained by iron chloride and PEG 8000 precipitation were pooled together and bacteriophages were further concentrated by ultracentrifugation (Optima XE90, Beckman-Coulter, rotor type 90 Ti, Beckman-Coulter) at 77,000 rcf for 1 h at 4°C (Szpara et al., 2011). A P1 reference bacteriophage NC_005856.1 was used as a positive control for DNA extraction. The pellet was resuspended in 1 ml of supernatant and the total DNA was extracted according to the protocol published by Thurber et al. (2009). Using the Qubit dsDNA HS assay kit (Life), the extracted DNA was quantified at 0.2 ng DNA.

### Library preparation and sequencing

To prevent amplification biases, yet obtain enough DNA for Ion Torrent sequencing, 43.2 ng of 16S ribosomal DNA of “*Candidatus* K. stuttgartiensis” was added to 0.1 ng of viral DNA (Cremers et al., in preparation). The total DNA was sheared for 6 cycles using the Bioruptor (Diagenode, Liege, Belgium; 1 min on, 1 min off) and prepared according to manufactures protocol (IonXpress Plus gDNA fragment library preparation Rev C.0, Life). The sample was sequenced using an Ion Torrent Personal Genome Machine (Life) on a v318 chip following manufacturer's protocol.

### Bioinformatics

#### Contig assembly

The sequencing reads (4,334,460) were trimmed with default quality settings (size 25–375 bp) using CLC genomics workbench (CLCbio, Aarhus, Denmark) and filtered to remove 16S ribosomal *K. stuttgartiensis* DNA (~87.6%) and *M. oxyfera* genomic DNA (~0.08%). The remaining reads were assembled using SPAdes (v.3.5.0), and ESOM (Ultsch and Mörchen, 2005; Dick et al., 2009) was used to validate the consistency of the contig tetranucleotide profiles (Supplementary File 1). This resulted in 2094 assembled contigs with a total length of 1.87 Mbp, including the five proposed genome sequences (Supplementary Files 2A–C).

#### Annotation of genes on the bacteriophage contigs

Putative ORFs (open reading frames) were predicted and automatically annotated on all contigs (Supplementary File 3) by using Prokka (Seemann, 2014). The five longest and putative complete viral genomes were selected for manual annotation, and visualized by ARTEMIS (Rutherford et al., 2000). The automatic annotation of each putative ORF was manually curated using BLASTp (Johnson et al., 2008). An *e* < 10^−3^ was chosen as a cut-off for annotation of genes and domains. The amino acid sequences were also analyzed for the presence of conserved domains consulting: InterProScan (Jones et al., 2014), PROSITE (De Castro et al, 2006), Motif Scan (Pagni et al., 2007), Pfam (Finn et al., 2016), and SMART (Letunic et al., 2014). For each putative ORF, percentage identity and overlap to the first annotated/validated hit obtained through BLASTp were calculated using Pairwise Alignment (Wu et al., 2003). Classification of annotated genes was performed according to the following criteria. Hypothetical protein; >20% identity to hypothetical protein, conserved hypothetical protein; >30% identity to hypothetical protein (similar length), similar to; >20% identity to known protein (similar length), strongly similar to; >40% identity to known protein (similar length). ORFs that contained conserved domains (but no annotated Blast hits) or (strong) similarity to a known protein but different length were described as putative [protein name].

To assess the presence of known viruses and bacteria in the viral metagenome, all contigs obtained with SPAdes and their predicted ORFs were compared against the nr databases from NCBI (using BLASTn and BLASTp, respectively) and the Earth's virome database (Paez-Espino et al., 2016; Supplementary File 4).

#### Accession numbers

The viral genomes are available from GenBank under accession numbers, KX853510, KX853511, KX853512, KX853513, KX853514 for Genomes 1–5, respectively. The remaining contigs are submitted under number MKFH00000000.

### Predicting which of the sequenced bacteriophages infects *Methylomirabilis* sp.

Computational phage-host prediction signals (Edwards et al., 2015) were calculated to identify which of the 2094 assembled contigs belonged to the bacteriophage infecting *Methylomirabilis*. First, genetic homology between the *Methylomirabilis* genomes (*Methylomirabilis oxyfera*; Ettwig et al., 2010 and a new *Methylomirabilis* species; Guerrero et al., in preparation) and the viral contigs was determined by using blastn and tblastx 2.2.30+ with default settings and *E* ≤ 10^−5^ (Camacho et al., 2009), and the total bitscore for each viral contig was recorded. Second, both *Methylomirabilis* genomes were checked for CRISPR sequences using CRISPRfinder (http://crispr.u-psud.fr/Server/) and CRASS (Skennerton et al., 2013). The resulting spacer sequences were subsequently cross-referenced with the viral genomes and contigs from the SPAdes assembly using CRISPRtarget (Biswas et al., 2013). Third, similarity in oligonucleotide usage was calculated as 1—E, where E is the Euclidean distance between the k-mer profiles of each viral contig and the *Methylomirabilis* genomes (*k* = 2, 4, 6).

## Results

### Bacteriophage population in a *Methylomirabilis* bioreactor enrichment culture

**Figure 1:**
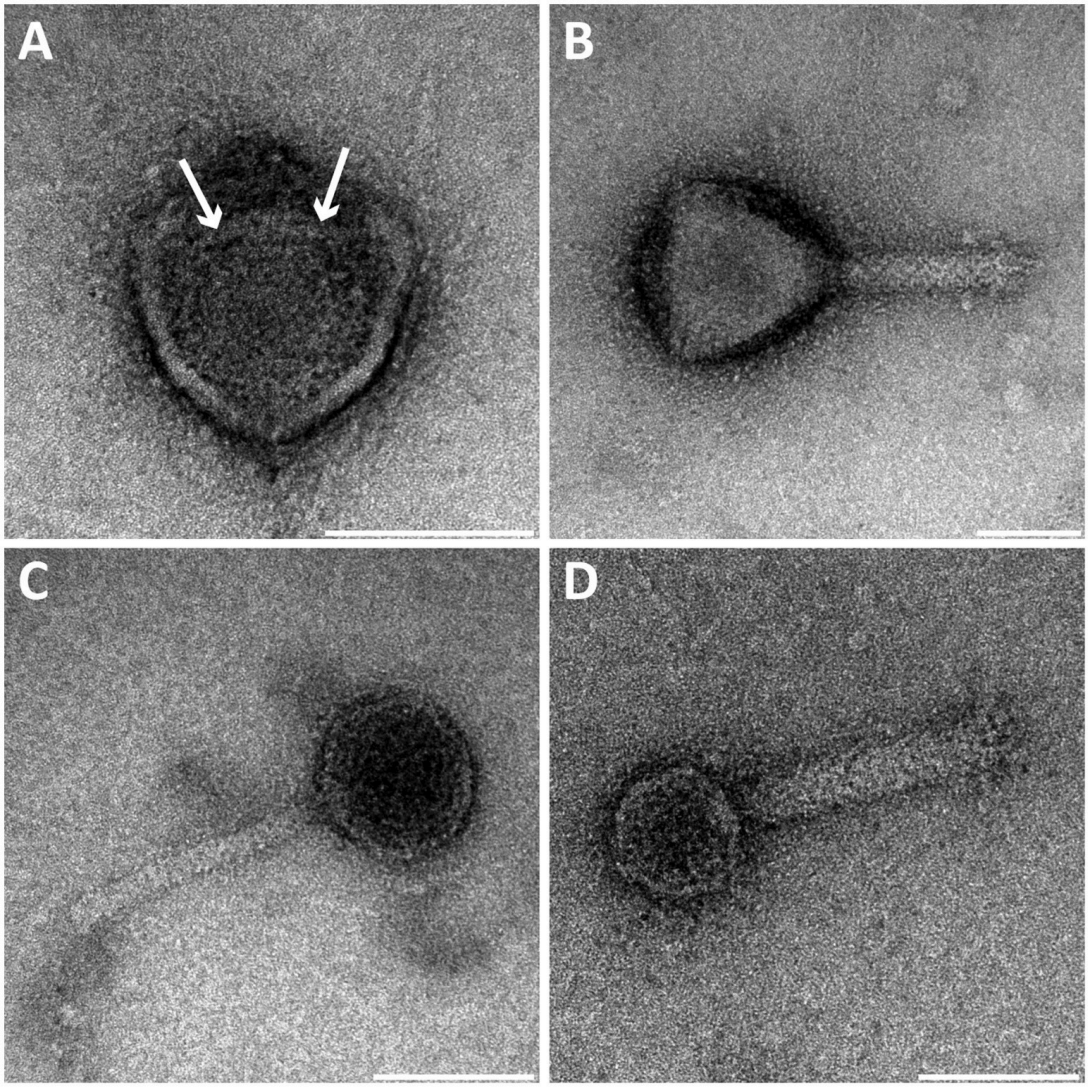
**Transmission electron micrographs of negative-stained bacteriophages present in the ***Methylomirabilis*** bioreactor enrichment culture**. Four viral morphotypes were observed. **(A)** Viral morphotype with putative icosahedral symmetry of the head (~71 nm diameter), without a tail or other appendages but with a central round internal core (~25 nm diameter, see arrows). **(B–D)** Three additional viral morphotypes all with a tail and a putative icosahedral symmetry of the head (~87, ~50, and ~41 nm diameter respectively). Scale bars; 50 nm.

We investigated the bacteriophage population in a bioreactor containing an enrichment culture of the anaerobic methanotroph *Methylomirabilis* sp. (Table 1). Several free bacteriophages with different morphologies were observed using negative staining. Among these, four morphotypes were identified. All bacteriophages had putative icosahedral capsid symmetry and three of them possessed a tail. Capsids and tails varied in diameter and length, thereby allowing a clear distinction between the morphotypes (Figure 1).

**Table 1:** **Properties of the 4 viral morphotypes observed in the ***Methylomirabilis*** enrichment culture**.

**Bacteriophage**	Capsid size (nm)^*^	**Capsid symmetry**	Tail length (nm)^*^	Tail width (nm)^*^	**Morphotype**
Figure 1A	~66	Icosahedral	–	–	Non tailed virus
Figure 1B	~87	Putative icosahedral	~90	~21	Myoviridae-like
Figure 1C	~50	Putative icosahedral	~87	~8.5	Myoviridae-like
Figure 1D	~41	Putative icosahedral	~91	~16	Myoviridae-like

* *All reported measures were obtained from negative-stained samples*.

### Bacteriophage infection in *Methylomirabilis* cells

**Figure 2:**
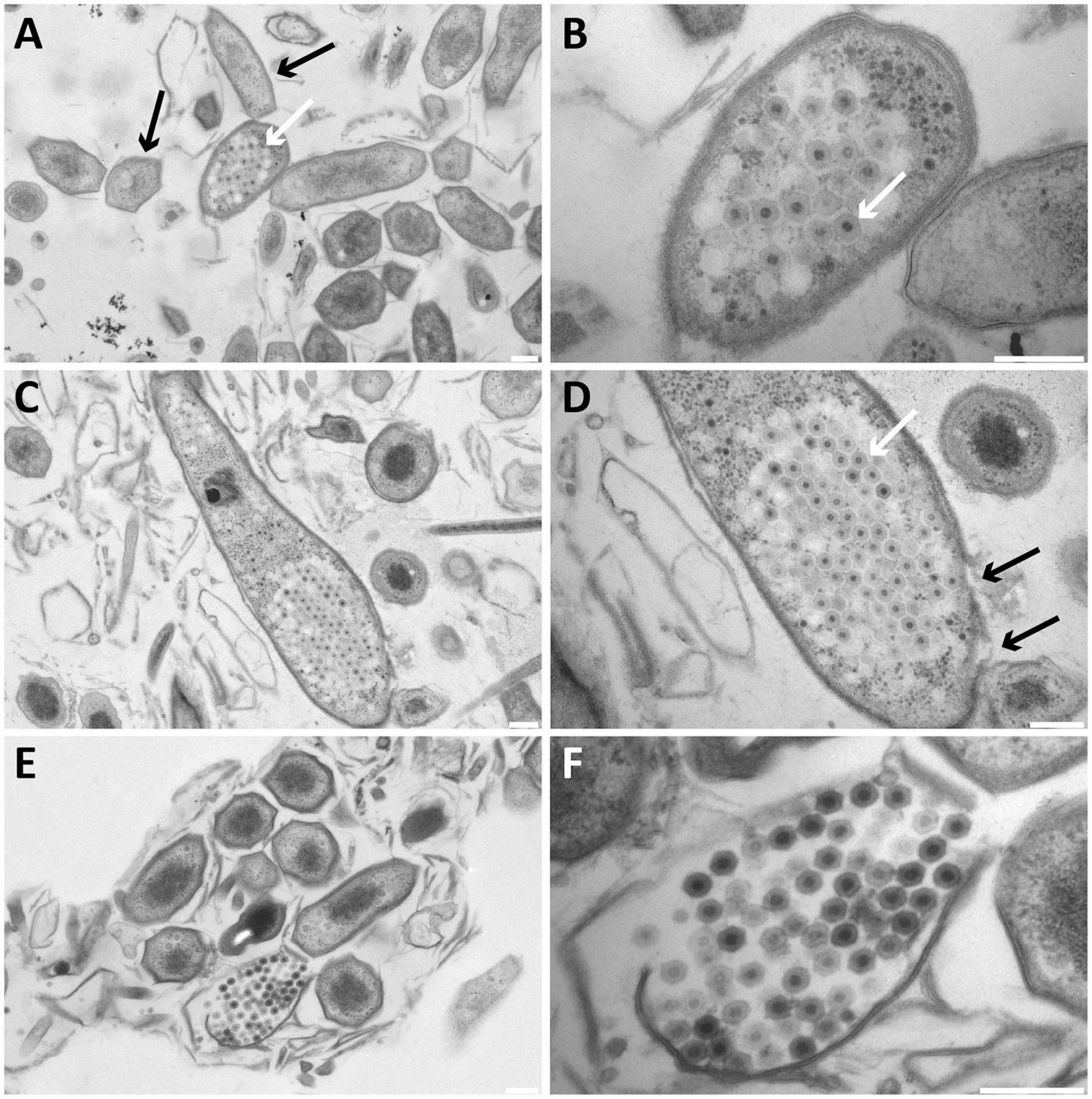
**Transmission electron micrographs of high-pressure frozen, freeze-substituted, resin-embedded, and thin-sectioned ***Methylomirabilis*** cells taken from a bioreactor enrichment culture. (A,B)** Infected *Methylomirabilis* cells (white arrow in **A**) are in clusters among non-infected cells (black arrows in **A**). The bacteriophage (white arrow in **B**) has a hexagonal shape and an internal electron dense core. **(C,D)** The bacteriophages are organized in a highly packed formation (white arrow in **D**). The replication and assembly of bacteriophages causes the *Methylomirabilis* cell to swell and eventually the cell wall breaks (black arrows in **D**). **(E,F)** Lysed *Methylomirabilis* cell releasing the viral progeny. **(B,D,F)** are enlargements of **(A,C,E)**, respectively. Scale bars; 200 nm.

**Figure 3:**
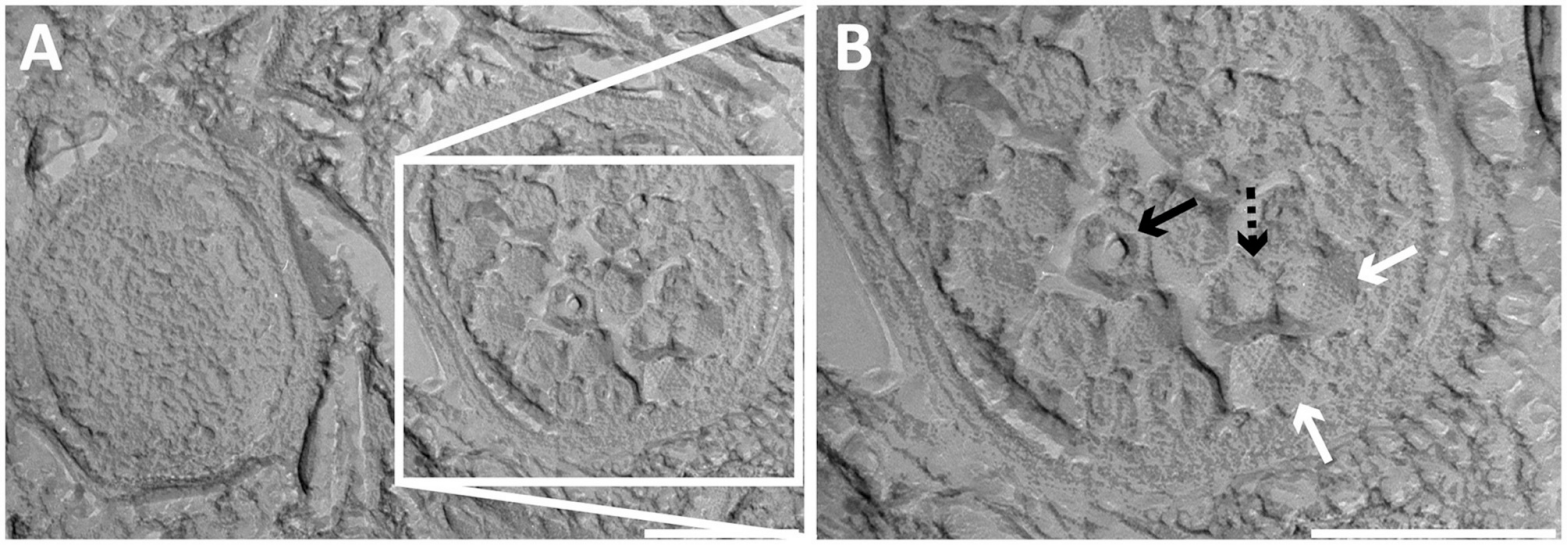
**Transmission electron micrographs of high-pressure frozen and freeze-etched ***Methylomirabilis*** cells taken from a bioreactor enrichment culture. (A)** Cross-section of a non-infected (left) and infected (right) *Methylomirabilis* cell. **(B)** The bacteriophages (white arrows) contain a proteinaceous capsid. The capsid is made of triangular faces built by capsomeres. Concave (dashed black arrow) and convex (black arrow) printing of the internal core is visible in two of the viral particles. Scale bars; 200 nm.

In addition to free bacteriophages, the microbial population was investigated for phage infection using thin sections, freeze-etching, and electron tomography. Thin sections of high-pressure frozen, freeze-substituted, and epon embedded biomass samples showed a lytic phage infection in *Methylomirabilis* cells (Figure 2). The infection rate (amount of infected *Methylomirabilis* cells in the total *Methylomirabilis* population) was calculated to be 2.3%. The infection did not affect bioreactor performance with respect to activity (methane and nitrite consumption). The intracellular bacteriophages had a hexagonal capsid (ca. 55 nm wide, vertex-to-vertex), indicating an icosahedral 3D symmetry. The capsid contained an electron dense and round central core (ca. 20 nm diameter), probably enclosing the genetic material. The bacteriophages were present both outside and inside the *Methylomirabilis* cells. Infected *Methylomirabilis* cells displayed different stages of bacteriophage assembly. In addition to completely assembled bacteriophages, entities were observed inside the cells that contained only the central core, i.e., the capsid was not yet assembled. As more and more bacteriophages were assembled, they were organized in a highly packed formation within the cell (Figures 2C,D). This caused the infected cell to swell approximately 1.9x in size compared to a not infected cell [area measured on TEM sections based on 10 infected (0.44 ± 0.2 μm^2^) and 10 not infected cells (0.23 ± 0.06 μm^2^)]. Afterwards the infected cell lysed thereby releasing the bacteriophages (Figures 2E,F). Comparing the intracellular *Methylomirabilis* bacteriophage to the free bacteriophages observed in the bioreactor enrichment culture using negative staining, its morphology resembled that of the bacteriophage depicted in Figure 1A based on size and symmetry. Freeze-etching of infected *Methylomirabilis* cells revealed the surface of the bacteriophage capsid (Figure 3). The capsomeres, the building subunits of the capsid, were arranged in triangular faces that constitute the icosahedral symmetry of the proteinaceous capsid.

**Figure 4:**
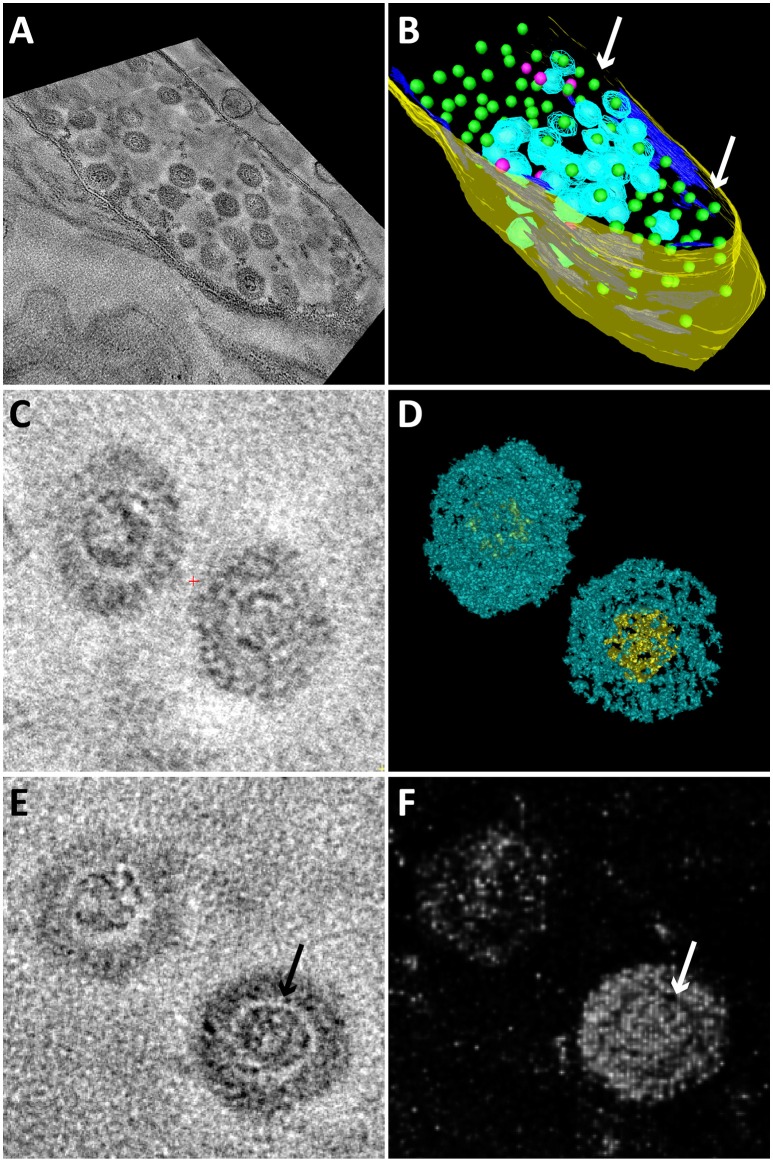
**Snapshots of electron tomograms and models of free and intracellular bacteriophages infecting ***Methylomirabilis*** cells**. Tomogram **(A)** and model **(B)** of an infected *Methylomirabilis* cell. Most bacteriophages have the capsid (blue) assembled around the electron dense core (green). Some bacteriophages are still in the process of assembly and only consist of the electron dense core (pink). The cell is swollen and the cytoplasmic membrane (dark blue) is broken at many places (arrows). The cell wall (yellow) is still intact. All green electron dense cores were surrounded by a capsid, but not all capsids were modeled for reasons of clarity. Tomogram **(C)** and isosurface density model **(D)** of two free bacteriophages showing the icosahedral capsid (blue) and electron dense core (yellow). Tomogram **(E)** and Chimera model **(F)** showing two free bacteriophages. The electron dense core is enclosed by a putative membrane (arrows).

Electron tomography was used to study the 3D structure of the bacteriophage and the infected *Methylomirabilis* cells (Figure 4). 3D reconstruction and modeling showed that the cytoplasmic membrane of infected cells was broken at many places while the cell wall was often still intact (Figures 4A,B and Supplementary Movie 1). Inside infected cells, both completely assembled bacteriophages were present as well as incompletely assembled bacteriophages (with only the electron dense core visible—no capsid yet). 3D reconstruction and modeling of free bacteriophages suggested the presence of a putative internal membrane surrounding the electron dense core (Figures 4C–F). In addition, no tail was observed on the free bacteriophages.

### Viral metagenome of the bacteriophage population in the *Methylomirabilis* bioreactor enrichment culture

**Figure 5:**
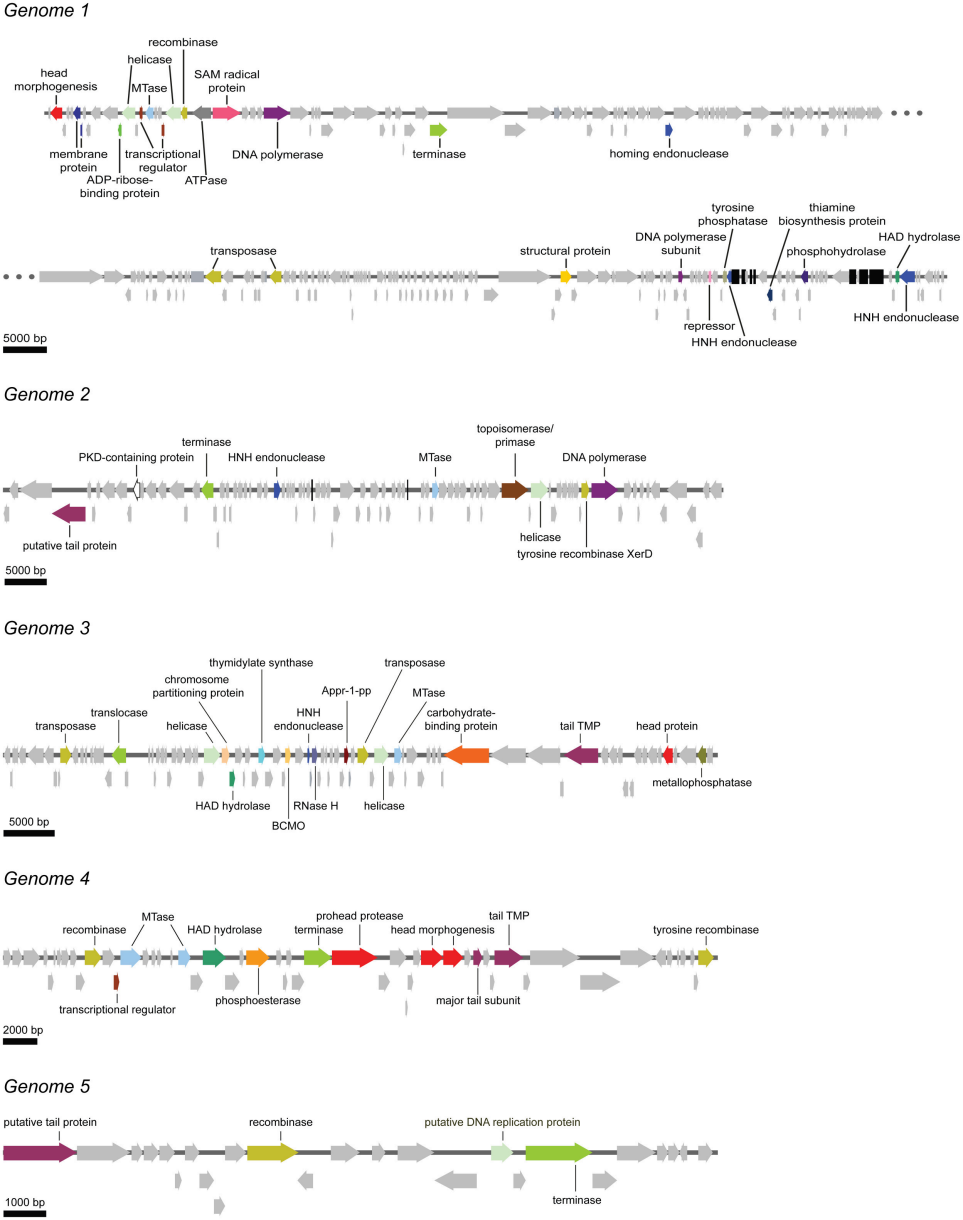
**Schematic overview of the five putative complete viral genomes retrieved from the viral metagenome of the bacteriophage population in the ***Methylomirabilis*** bioreactor enrichment culture**. Arrows with the same color represent genes encoding for the same putative functional category. Light gray arrows indicate hypothetical proteins. Black bars in genome 1 and genome 2 represent tRNAs. Appr-1-pp, ADP-ribose 1-phosphate phosphatase; BCMO, β-carotene 15, 15′-monooxygenase; HAD hydrolase, haloacid dehalogenase; MTase, methyltransferase; PKD-containing protein, polycystic kidney disease-containing protein; RNase H, ribonuclease H; tail TMP, tail tape measure protein.

To characterize the bacteriophage population present in the *Methylomirabilis* bioreactor enrichment culture, the total viral DNA was extracted from the bioreactor and the effluent and sequenced. After assembly, 2094 contigs were obtained (Supplementary Files 2A,B). The five longest and putative complete viral genomes (Table 2) were selected for manual annotation (Figure 5 and Supplementary Files 5A–E). Four of these contigs (197, 85, 71, 16 kb) contained identical sequences at both ends, indicating a full genome and a circular genomic arrangement. The 41 kb contig did not have overlapping end sequences, possibly representing a linear genome.

**Table 2:** **Genomic characteristics of the five putative complete viral genomes retrieved from the viral metagenome of the bacteriophage population in the ***Methylomirabilis*** bioreactor enrichment culture**.

**Genome**	**Genomic arrangement**	**Genome length (kb)**	**Number of reads**	**Depth**	**GC content (%)**	**# ORFs**
1	circular	197	254,185	320.1	62.9	280
2	circular	86	213,396	266.8	54.1	103
3	circular	71	320,546	41.2	54.3	102
4	linear	41	672,323	86.4	57.2	54
5	circular	17	173,132	219.0	67.8	25

#### Phage genome 1

In this genome, one head morphogenesis protein was found, involved in initial stages of head assembly (Hsiao and Black, 1978; Leiman et al., 2003). DNA replication enzymes are represented by one DNA polymerase and one DNA polymerase subunit. In addition, the two helicases may also take part in the replication process. The DNA packaging machinery in genome 1 includes three terminases. Compared to the other five annotated genomes, genome 1 was the only annotated genome that does not contain a clear hit to a tail protein. In addition, genome 1 encodes for one putative ATPase, maybe involved in DNA packaging (Kondabagil et al., 2006), and two ORFs with a blast hit against a membrane protein. The genome encodes no less than 41 tRNAs. The acquisition of these tRNAs from the host might function in providing the phage with a similar GC content as the host, thereby facilitating the recruitment of the host DNA replication enzymes (Bailly-Bechet et al., 2007; Enav et al., 2012).

#### Phage genome 2

Genome 2 encodes for one putative tail protein. No capsid-related genes could be annotated. The DNA replication machinery in genome 2 consists of one DNA polymerase, one helicase and one primase. Interestingly, a PKD (polycystic kidney disease) domain-containing protein was also annotated. These proteins may have a function in mediating phage-host contact (Fraser et al., 2006, 2007; Sathaliyawala et al., 2010) and BAM (bacteriophage adherence to mucus) mechanisms (Barr et al., 2013).

#### Phage genome 3

Genome 3 contains one tail TMP (tape measure protein) and one head protein. Two helicases and one RNase (ribonuclease) H are the only three annotated genes that could be part of the DNA replication machinery. One chromosome partitioning protein similar to ParB was found in this genome. A ParB protein is also encoded by the *E. coli* phage P1, which can be present in the cell as a plasmid or integrated in the bacterial DNA (Abeles et al., 1985). The presence of a ParB-like protein in genome 3 may indicate that this bacteriophage might also be capable of integrating in the host genome or forming a plasmid. A putative phage-host interaction protein was also identified. This is a carbohydrate-binding protein with an Ig-like fold. Carbohydrate-binding domains have been found on tail spike proteins (Andres et al., 2010) and head spikes (Westbye et al., 2016), in both cases mediating the recognition of the target polysaccharides in the initial steps of infection.

#### Phage genome 4

Genome 4 has three proteins involved in head assembly, being a prohead protease and two head morphogenesis proteins. Next to them in the genome two tail proteins are located (one tail TMP and one major tail subunit).

#### Phage genome 5

Genome 5 encodes for a putative tail protein but no head proteins could be annotated. Most of the ORFs in this small genome are hypothetical proteins.

All annotated genomes show the putative ability to establish lysogeny. One transposase was found in genomes 2 and 5, two transposases in genomes 3 and 4 and three transposases in genome 1. These enzymes mediate unidirectional and site-specific recombination between two DNA sequences, one on the viral genome and one on the host genome (Groth and Calos, 2004). All genomes encode for one or two terminases (or translocase). These enzymes are responsible for packaging the genome inside the capsid (Catalano, 2000; Duffy and Feiss, 2002). HNH endonucleases found in double copy in genome 1 and in single copy in genomes 2 and 3 may also be involved in cutting the genome while DNA packaging is taking place (Moodley et al., 2012; Kala et al., 2014). Methyltransferases were also frequently found in single copy (genomes 1, 2, and 3) and double copy (genome 4). In bacteriophages methyltransferases are often thought to protect the viral genome from degradation by host-encoded edonucleases. However, few studies hypothesize a role in DNA replication and regulation both in lysogenic and lytic cycles (Murphy et al., 2013).

### Genome prediction of the *Methylomirabilis*-infecting bacteriophage

A main focus of the viral metagenomics in this study was to genomically identify the bacteriophage infecting *Methylomirabilis* cells (Figures 2–4). Based on the five viral genomes and over two thousand contig sequences obtained above, it is not directly clear which cellular hosts the recovered bacteriophages infect. Thus, to address this question we applied several computational approaches to predict this phage-host relationship from sequence signals (Edwards et al., 2015) that linked the assembled viral contigs to both the original genome and that of the new *Methylomirabilis* species. As described in Edwards et al. (2015), we calculated three main categories of phage-host prediction signals: genetic homology, similarity in CRISPR spacers, and similarity in oligonucleotide usage (Supplementary File 6). Genetic homology is expected to occur between the viral and bacterial genome sequences due to processes including transduction. This was identified at the nucleotide level (blastn, *E* ≤ 10^−5^) and at the protein level (tblastx, *E* ≤ 10^−5^), the former detecting only five short fragments, the latter detecting many more contigs including the long genomes 1–4. However, in all cases the matches between the bacteriophages and the *Methylomirabilis* genomes were based on relatively short, scattered regions of similarity with low sequence identity. As it was also showed by Edwards et al. (2015), the similarities in oligonucleotide usage profiles (*k* = 2, 4, 6) are not strong as phage-host predictors.

In the previously described *M. oxyfera* genome (Ettwig et al., 2010) one CRISPR array was present, which contained 23 spacers (Supplementary File 7). In the genome of a new *Methylomirabilis* species (Guerrero et al., in preparation) derived from the reactor from which the virome was sequenced, two CRISPR arrays were observed; one of which contained 26 spacers and the other one six spacers (Supplementary File 7). No significant nucleotide similarity was detected between these spacers and any of the 2094 possible viral contig sequences with fewer than 11/37 mismatches, which means these hits are indistinguishable from random noise (Edwards et al., 2015).

We also looked for signs of lysogeny in the *M. oxyfera* metagenome (Ettwig et al., 2010) and the new *Methylomirabilis* sp. metagenome (Guerrero et al., in preparation). The viral contigs were compared using BLASTn searching for contigs that were partly viral and partly bacterial. However, no such contigs were present in neither of the two metagenomes.

## Discussion

We analyzed the bacteriophage community in a bioreactor enrichment culture of the anaerobic methane oxidizing bacterium *Methylomirabilis* sp. Four different bacteriophage morphologies were observed inside the bioreactor system, one of which was observed to infect *Methylomirabilis* cells. From the viral metagenome data at least five putative complete viral genomes were assembled. Based on bioinformatics including CRISPR analyses, we could not identify the viral genome of the *Methylomirabilis* infecting bacteriophage.

Viral metagenome analysis of bioreactor enrichment cultures is challenging. In this case, the viral abundance was low and it required 13 L bioreactor material to obtain 0.2 ng viral DNA. We used a newly developed method for obtaining the (dsDNA) viral metagenome from the low amounts of viral DNA (Cremers et al., in preparation). In the end, 2094 contigs were assembled. Among these we identified five putative complete viral genomes. The annotation of these five genomes resulted in many hypothetical proteins. Metagenomics reads are often short and will not lead to improved annotation if viruses are not isolated and further experiments performed. Therefore, the viral sequences in the databases represent only a small fraction of the entire viral sequence space (Mokili et al., 2012; Dutilh, 2014). Nevertheless, we could in most cases identify putative genes involved in DNA processing and tail and capsid formation.

Electron microscopy analyses showed a bacteriophage infecting *Methylomirabilis* cells at a relatively low infection rate of 2.3%. The bacteriophage performed a lytic cycle as bacteriophages were replicated inside the host cell, causing the host to swell. In the end, the host cell lysed and released the viral particles. The bacteriophage had a putative icosahedral capsid and no tail. Inside the bacteriophage, a round electron dense core was visible, which most probably represented the highly packed genome. Electron tomography suggested the presence of a putative internal membrane, which appeared as an electron-light area surrounding the genome. Such an electron-light structure around a viral genome has not been observed for the other described morphotypes, therefore this could be an indication that this genome was enclosed by a structure, most likely a lipid membrane, as it was also shown for bacteriophages with similar morphology (Harrison et al., 1971; Mindich et al., 1982). Inside the infected *Methylomirabilis* cells incompletely assembled bacteriophages were observed constituted by only the electron dense core. The assembly of this bacteriophage might thus proceed by the following steps: membrane formation, packaging of the genome, and assembly of the capsid.

The presence of an internal membrane surrounding the viral genome has so far only been described for two bacteriophage families: *Tectiviridae* (four phages described and PRD1 as type species) and *Corticoviridae* (one phage described; PM2; King et al., 2012). These bacteriophages infect Gram-negative bacteria, have a relatively small dsDNA genome [10 kb circular, *Corticoviridae* (Männistö et al., 1999); 15 kb linear, *Tectiviridae* (Bamford et al., 1991)] and have an icosahedral capsid [66 nm from facet to facet for *Tectiviridae* (Abrescia et al., 2004) and 55 nm from facet to facet for *Corticoviridae* (Kivelä et al., 2002)] with no tail. It was described that these bacteriophages obtain their internal membrane from the host cytoplasmic membrane using so called membrane proteins (Espejo and Canelo, 1968; Mindich et al., 1982). Also in the present study, the cytoplasmic membrane of infected *Methylomirabilis* cells was not intact anymore as was apparent from 3D electron tomography models. The similar ultrastructure of the *Methylomirabilis* bacteriophage to the *Corticoviridae* and *Tectiviridae* might indicate that it belongs to one of these families.

By using a bioinformatics approach, we tried to predict which viral contigs (2094) assembled from the viral metagenome data belonged to the *Methylomirabilis*- infecting bacteriophage. However, we were not able to obtain any convincing result. Homology searches yielded only short spurious hits, while the oligonucleotide usage profiles were too broad and did not provide a specific signal to any one of the longer contigs. In addition, the CRISPR arrays that were identified in the *Methylomirabilis* genomes did not have a clear hit to any of the assembled bacteriophage contigs. There are several possible explanations for the latter. First, since bacteriophage genomes with high similarity to one or more spacers in a CRISPR array are precluded from infection, CRISPR spacers are a focal point of positive selection in bacteriophage genome sequences, because the only bacteriophages that survive are the ones with mutations in the protospacer (Sun et al., 2013; Paez-Espino et al., 2015). Indeed, the closest match between CRISPR spacer and viral contigs already showed 11 mismatches. Second, the spacer composition of CRISPR arrays in a bacterial community can be very volatile due to ecological selection for bacterial strains with spacers acquired from the current bacteriophages in the environment (Rho et al., 2012; Koskella and Brockhurst, 2014). Thus, even though the *Methylomirabilis* genomes that were mined for CRISPR spacers were obtained from the same bioreactor (Ettwig et al., 2010; Guerrero et al., in preparation), not a single one of the CRISPR spacers was found in common. Taken together, computational phage-host signals did not result in a strong candidate for the bacteriophage infecting *Methylomirabilis* in this bioreactor.

We also investigated signs of lysogeny by in depth analyses of the *M. oxyfera* metagenome (Ettwig et al., 2010) and the new *Methylomirabilis* sp. metagenome (Guerrero et al., in preparation), looking for contigs that were partly bacterial and partly viral. However, no such sequences were detected. This could indicate that the bacteriophage does not perform a lysogenic cycle but only a lytic one. In the end, we can only speculate about which viral genome might belong to the bacteriophage observed to infect the *Methylomirabilis* cells. Unfortunately none of the five putative complete viral genomes matched all (indirect) criteria. The bacteriophage morphology (icosahedral capsid, no tail, genome surrounded by putative internal membrane) indicated a relation to the *Tectiviridae* and *Corticoviridae* families that have a relatively small genome size, although the bacteriophage could also belong to a yet-undiscovered family. The only small (complete) genome (genome 5–17 kb) contained a putative tail protein indicating that most likely it was not our tailless bacteriophage. There was one genome without an annotated tail gene but it was fairly large (genome 1–197 kb) which did not fit to the described genome size in these families. Other bacteriophages with an internal membrane and a genome size bigger than 15 kb have been described (Aalto et al., 2012; Rissanen et al., 2013) but they are not yet assigned to a viral family.

In our study we focused on DNA-containing viruses. One explanation why the applied bioinformatic approaches could not find any phage-host interaction signals could be because the *Methylomirabilis*-infecting bacteriophage might have a ssDNA or an RNA genome. However, ssDNA viruses and especially RNA viruses comprise only a small minority among prokaryotic viruses, dominated instead by dsDNA viruses (Koonin et al., 2015). Also, especially considering the low yield of viral DNA, we might not have retrieved the entire bacteriophage population from the bioreactor. Not until methods as phageFISH (Allers et al., 2013) are optimized to be used for non-model systems it remains difficult to link a phage to a host through non bioinformatic methods.

The *Methylomirabilis* infection rate was observed to be relatively low (2.3% of all infected *Methylomirabilis* cells) at different time points throughout the period of ~1 year and did not affect bioreactor performance with respect to activity and growth. Indeed, the bacteriophage did not kill the *Methylomirabilis* population. Metagenomics analysis of the enrichment culture indicated the presence of a new *Methylomirabilis* species (Guerrero et al., in preparation), which was not present in the bioreactor when the genome of the original *M. oxyfera* species was published (Ettwig et al., 2010). An interesting speculation could be that the bacteriophage was specific for only the original *M. oxyfera* species (Ettwig et al., 2010) and in the end instigated the shift from the original to the new *Methylomirabilis* species. Indeed currently, the infection has almost disappeared from the enrichment culture, the published *M. oxyfera* 16S rRNA gene (NCBI, FP565575) cannot be detected anymore and the enrichment culture is dominated by the new *Methylomirabilis* species.

Bacteriophages can have profound effects on bacterial populations also in bioreactor enrichment systems. This has to be taken into account in the application of microorganisms in, for example, wastewater treatment systems or other industrial applications. In general, how bacteriophages can shape environmental or industrial microbial communities is still largely unexplored and is an exciting topic for further study.

## Author contributions

LV and MJ designed the project. LG and LV designed the experiments. SG maintained and enriched the *Methylomirabilis* culture. LG and RM performed all TEM related experiments. LG performed the concentration of viral particles and DNA extraction. GC performed the library preparation and sequencing. GC, BD, and HO designed, performed and analyzed the bioinformatics research. LG manually annotated the complete viral genomes. LV supervised the research. LG and LV performed data analysis, data interpretation and wrote the manuscript with input from GC, RM, SG, BD, MJ, and HO.

### Conflict of interest statement

The authors declare that the research was conducted in the absence of any commercial or financial relationships that could be construed as a potential conflict of interest.
