# Supplementary material for: Ultrastructure and Viral Metagenome of Bacteriophages from an Anaerobic Methane Oxidizing Methylomirabilis Bioreactor Enrichment Culture
Source: Front Microbiol. 2016 Nov 8;7:1740. doi: 10.3389/fmicb.2016.01740 (PMC5099504; doi:10.3389/fmicb.2016.01740)
Supplement: Supplementary file 2 [file DataSheet1.DOCX]

**Supplementary File 1**. ESOM mapping of the results after assembly with SPAdes. Each dot represents a DNA fragment (size between 1 kb and 1.5 kb) and the background represents the similarity between the DNA fragments. DNA fragments with a similar tetranucleotide profile are plotted more closely together than dissimilar DNA fragments. Genomes 1 to 5 were highlighted in different colors: genome 1 = yellow, genome 2 = dark blue, genome 3 = dark pink, genome 4 = green, genome 5 = light blue. Scale bar indicates similarity of the tetranucleotide composition.

**Supplementary File 2A**. Viral metagenome of the bacteriophage population in the *Methylomirabilis* bioreactor enrichment culture. After reassembly with SPAdes, 2094 contigs were obtained. For each contig, the vertical average coverage (depth), the GC content, contig length, accession number in NCBI and the putative bacterial (in yellow) or eukaryotic (in red) origin of the sequences are given. Contigs marked as putative bacterial or eukaryotic share ≥90% sequence length with bacterial or eukaryotic sequences from the NCBI nucleotide database.

**Supplementary File 2B**. Viral metagenome of the bacteriophage population in the *Methylomirabilis* bioreactor enrichment culture. The assembly with SPAdes resulted in 2094 contigs. The nucleotide sequence of all contigs is given in FASTA format.

**Supplementary File 2C**. Coverage of the five genomes obtained after mapping viral reads to the assembled viral contigs. Mapping was performed with default settings and length_fraction = 0.5 similarity_fraction = 0.95 (CLC genomic workbench 8.5.1). The arrow indicates a possible breakpoint. The average coverage of each genome was: genome 1 = 320.1, genome 2 = 266.8, genome 3 = 41.2, genome 4 = 86.4 and genome 5 = 219.0.

**Supplementary File 3**. Automatic annotation of the viral metagenome of the bacteriophage population in the *Methylomirabilis* bioreactor enrichment culture. Summary of the automated Prokka annotation of all 2094 viral contigs (Prokka settings: --metagenome --kingdom Viruses). Numbers in the table indicate the number of genes with a given annotation according to Prokka. tRNA genes are summarized in one column.

**Supplementary File 4**. Results from BLAST comparison of the 2094 contigs against the NCBI nr database (nucleotide and protein), as well as the Earth’s virome database (Paez-Espino et al., 2016). All BLAST searches were performed with the CLC genomic Server 8.0.1. with default settings.

**Supplementary Files 5A-E**. Curated automatic annotation of the five longest and putative complete viral contigs from the viral metagenome of the bacteriophage population in the *Methylomirabilis* bioreactor enrichment culture. For each ORF (column A) the following characteristics are given: the start (column B) and end (column C) nucleotide (when in reverse direction the number is preceded by “C”), size in aa (column D), mass in kDa (column E), conserved domains (column F), sequence features (column G), first validated blast hit, organism, size of the hit in aa, E-value, pairwise alignment query:hit in aa, percent identity of the pairwise alignment (columns H-M respectively), and finally the putative function and final annotation of the ORF (columns N and O).

**Supplementary File 6**. Summary of the computational signals obtained to predict the genome of the *Methylomirabilis*-infecting bacteriophage. blastn: summed bitscore of blastn search of host genome against viral contig; tblastx: summed bitscore of tblastx search of host genome against viral contig; 2mers: Euclidean distance between 2mer profiles of host genome and viral contig; 4mers: Euclidean distance between 4mer profiles of host genome and viral contig; 6mers: Euclidean distance between 6mer profiles of host genome and viral contig; CRISPRs: summed bitscore of blastn-short search of viral contig against host CRISPR spacers.

**Supplementary File 7**. CRISPR characteristics of both the *M. oxyfera* genome (Ettwig et al., 2010) and the new *Methylomirabilis* sp. genome from Guerrero et al. (manuscript in preparation). The *M. oxyfera* genome contained one CRISPR-Cas system and the new *Methylomirabilis* sp. genome contained two. Proteins were compared using BLAST on the nr protein database.

**Supplementary Movie 1**. Movie of the electron tomogram and model depicted in Fig. 4A and B showing intracellular bacteriophages infecting a *Methylomirabilis* cell. Most bacteriophages have the capsid (blue) assembled around the electron dense core (green). Some bacteriophages are still in the process of assembly and only consist of the electron dense core (pink). The cell is swollen and the cytoplasmic membrane (dark blue) is broken at many places. The cell wall (yellow) is still intact. All green electron dense cores were surrounded by a capsid, but not all capsids were modelled for reasons of clarity.
