# Supplementary figures and images for: Ultrastructure and Viral Metagenome of Bacteriophages from an Anaerobic Methane Oxidizing Methylomirabilis Bioreactor Enrichment Culture
Source: Front Microbiol. 2016 Nov 8;7:1740. doi: 10.3389/fmicb.2016.01740 (PMC5099504; doi:10.3389/fmicb.2016.01740)

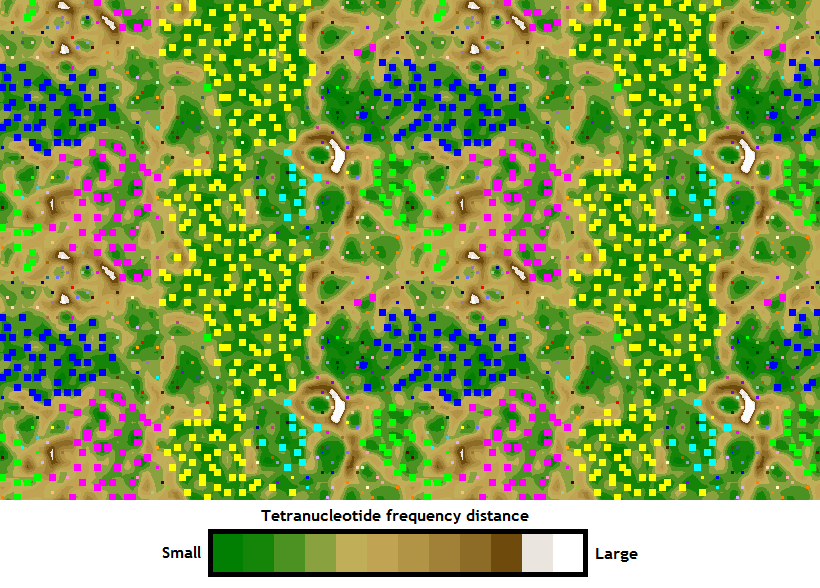

Supplement: Supplementary file 14 [file Image1.PNG]

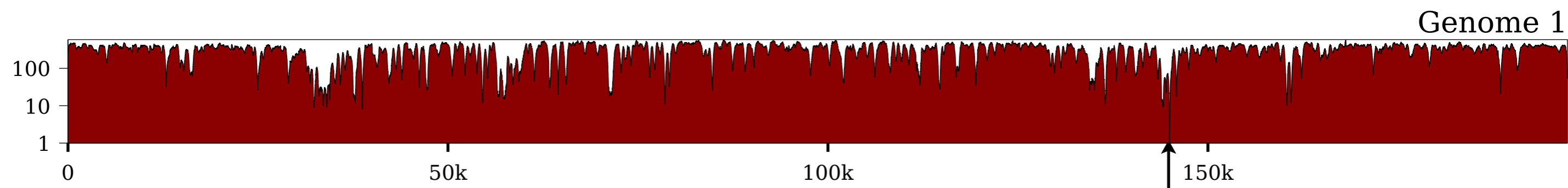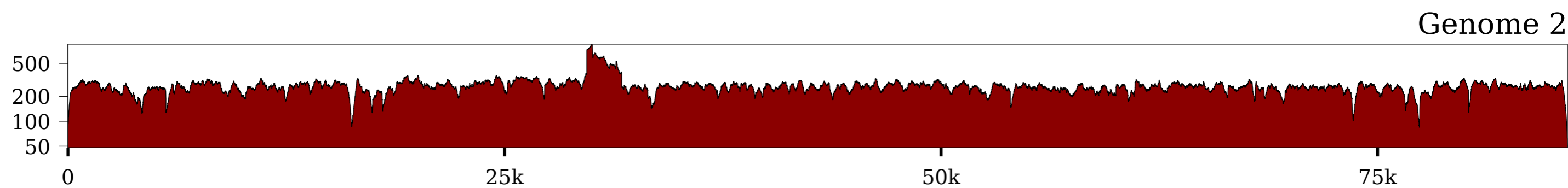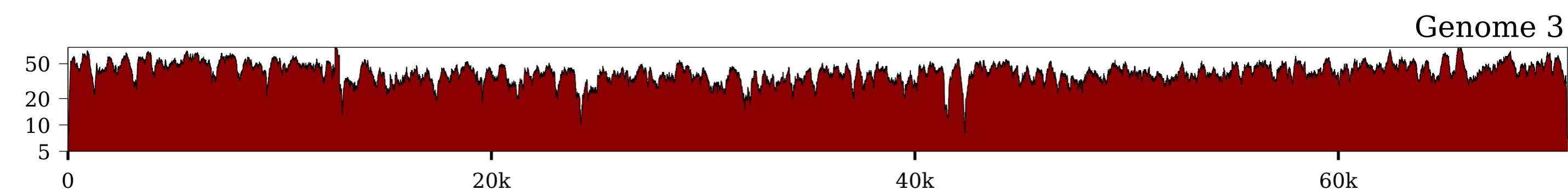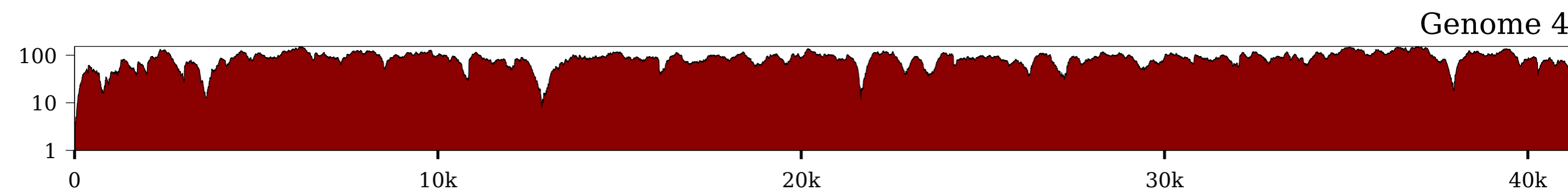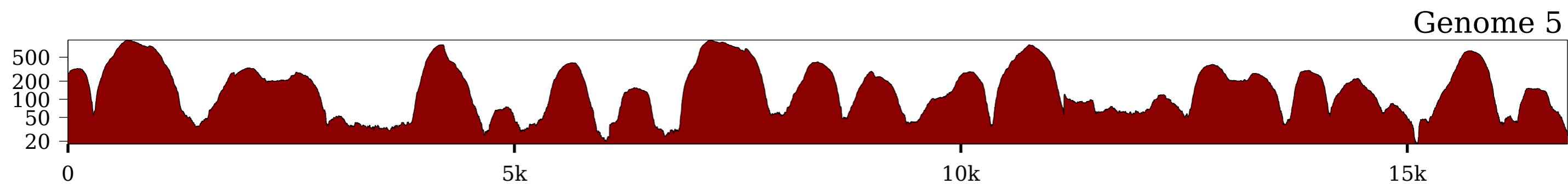

Basepairs

Supplement: Supplementary file 15 [file Image2.PDF]
